# Investigations into the Role of Friction for Rigid Penetration into Concrete-Like Material Targets

**DOI:** 10.3390/ma13214733

**Published:** 2020-10-23

**Authors:** Ningjing Jiang, Shufan Wu, Yile Hu, Zhongcheng Mu, Xiaofeng Wu, Wei Zhang

**Affiliations:** 1 School of Aeronautics and Astronautics, Shanghai Jiao Tong University, Shanghai 200240, China; ningjing@sjtu.edu.cn (N.J.); shufan.wu@sjtu.edu.cn (S.W.); yilehu@sjtu.edu.cn (Y.H.); 2 School of Aerospace, Mechanical and Mechatronic Engineering, University of Sydney, Sydney, NSW 2006, Australia; xiaofeng.wu@sydney.edu.au; 3 Harbin Institute of Technology, Harbin 150080, China; zhdawei@hit.edu.cn

**Keywords:** surface sliding friction, projectile acceleration, two-phase penetration, concrete-like targets

## Abstract

Currently, it appears that there is a lack of understanding related to the role of SSF, in the two-phase behavior of the deceleration history, which is an issue discussed recently in the impact dynamics field. This paper analytically and numerically focuses on the effect of SSF on the projectile deceleration characteristic of concrete-like targets. Firstly, the penetration process according to the two-phase feature of the projectile deceleration is revised, where analytical results indicate that the SSF has a phased feature corresponding to the two-phase behavior of the deceleration history. Furthermore, a series of numerical simulations are conducted to understand the role of SSF more clearly. Simulation results show a similar conclusion to the analyses of the two-phase penetration process; at the range below a certain critical striking velocity, adding friction can reproduce the experimental data; when exceeding the critical striking velocity, the simulated results without considering friction are closest to the experimental data. Hence, it could be gained that the role exchange between the SSF and the dynamic term contributes to the two-phase penetration behavior for concrete-like materials. This indicates that the sensitivity of SSF to the penetration process is one of the factors driving the two-phase feature.

## 1. Introduction

**Figure 1:**
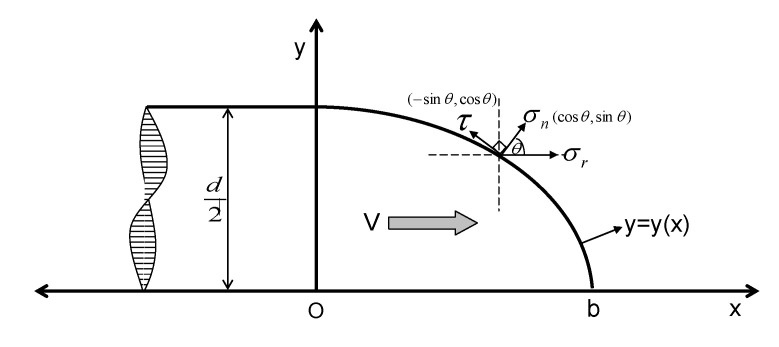
Force analytical graph of projectile nose [6].

Nondeforming projectile penetration into concrete-like materials has been studied for a wide range of applications with regard to civil and military shelters. Many studies related to the penetration of concrete-like materials dealt with the question of modeling and quantifying the force that resists the penetration [1]. However, due to the problems of complexity and the difficulty in performing measurements during the penetration process, analyses of the resisting force commonly weaken the Surface Sliding Friction (SSF) or simplify its effect [2,3,4,5]. In most cases, a Coulomb friction formula in the form of τ = μσn is accepted to describe the SSF, where τ and σn are, respectively, the stresses that are tangent and normal to the interface, and μ is the SSF coefficient, as shown in Figure 1 [6].

In several theoretical analyses and semiempirical equations, the SSF is usually neglected or a 0.02–0.2 static friction coefficient is used directly to gain good agreement with the experimental data [4,5]. Forrestal et al. [7,8,9,10,11,12,13] conducted a series of penetration experiments of different concrete-like materials, where evidences of friction and wear were proven through the observation of post-test projectile photographs.

On the other hand, Chen [14] firstly adopted the finite element code (a two-dimensional transient solid dynamics program) to investigate the SSF effect, where a constant (static) and velocity-dependent coefficients were introduced. Simulation results demonstrated that the application of both constant and velocity-dependent friction coefficients provided very similar results in terms of deceleration history and penetration depth. Moreover, simulation of a projectile with a conical nose, penetrating a rock target with an initial velocity of 520 m/s, yielded a mild plateau of the penetration deceleration, which was hardly affected by applying different types of friction coefficient. 

Meanwhile, some nonconstant friction algorithms were developed and applied in the analyses of force resisting the projectile penetration. Jones et al. [15] and Davis [16] developed one engineering model that considered dependence of the friction coefficient on the sliding velocity to obtain an optimal nose geometry. They concluded that the friction has a noticeable effect on the optimal nose geometry, which agreed with the observations of Forrestal et al. [4,17]. In these studies, a difference up to 25% was noticed when the friction coefficient was varied from 0.02 to 0.1. Furthermore, Ben-Dor et al. [18] developed localized interaction models with nonconstant friction for rigid penetrating impactors. Numerical results showed that the friction coefficient strongly affects the penetration depth in the case of high-speed penetration, in which the friction coefficient changes appreciably.

As discussed above, in a certain range of the initial striking velocity, consideration of the SSF between the projectile and the target is reasonable, where the coefficient of SSF has a monotonic decreasing correlation with the penetration velocity of the nondeforming projectile [14,15,16,18,19]. To our knowledge, the penetration depth and the deceleration history are the main criteria to evaluate the ability of the penetrator, especially for concrete targets, which requires the study of SSF behavior between projectile and target. Yet, the two-phase penetration behavior of the deceleration history does not consider the role of SSF, which was recently discussed in the Rosenberg and Dekel approach [20,21], Warren spherical cavity expansion (SCE) [22,23,24], Kong et al. extended SCE [25,26,27], and the DISCS model [28]. In order to ascertain the role of SSF for concrete-like materials subjected to impacts of rigid projectiles in the two-phase penetration behavior, the penetration process was studied via both analytical and numerical approaches. A series of two-dimensional (2D) numerical simulations, considering the dynamic damage feature of concrete-like materials, were carried out to clarify the SSF effect. The present study is helpful to better understand the two-phase behavior of the deceleration history of a projectile, and it further supports the reasonability of the two-phase penetration theory [6]. 

## 2. SSF Phase Characteristics

### 2.1. Two-Phase Behavior of the Deceleration History

The two-phase behavior of the deceleration history during rigid penetration was first introduced by Hill in his pioneering work during World War II [29]. He indicated that, in the penetration of ductile metals, there appears to be a threshold penetration velocity, below which the target always keeps full contact with the whole projectile whose nose smoothly connects its tail, and the resisting force is independent of the penetration velocity. This observation was explained by Hill to be a result of the absence of cavitation. When the penetration velocity exceeds the corresponding critical value, the contact feature is disappeared at a point of the projectile profile away from its bourrelet. Simultaneously, a cavitation larger than the projectile diameter is generated. The corresponding resisting force along the axial direction is included the penetration velocity.

Similarly, Yarin et al. [30] and Rubin [31,32] further supported Hill's viewpoint, where a rigid projectile with the shape of an ovoid of Rankine was investigated, and the incompressible elastic–perfectly plastic model was chosen to analyse the resisting force of the axial direction of the projectile. The analytical results indicated that the normalized value of the projectile resisting force is demarcated by a critical velocity.

Furthermore, rosenberg and Dekel [20] performed a series of 2D numerical simulations of aluminum and steel targets with different strengths. Simulated results indicated that for low striking velocities below a certain threshold striking velocity, the quasi-constant feature of the projectile deceleration is found. And it was found that the feature is depending only on the strength of target and the nose shape of rod. With the rise of the striking velocity until beyond the threshold striking velocity, the projectile deceleration becomes dependent on the striking velocity, which is attributed to the inertial response of target.

Afterward, in the arguments between Warren’s and Rosenberg and Dekel's work [21,22,24], Warren [24] also commented on the two-phase penetration phenomenon: “when the strength term overshadows the inertial term (the dynamic term in Equation (2)) the target inertia term can be neglected in the penetration model. However, when the strength does not overshadow the target inertia effects, then a target inertia term must be included”. This indicates that the sensitivity of the target inertia term to the penetration process is one of the reasons driving the two-phase feature.

Recently, Fan and Li [33] obtained the same conclusion for metal targets, using the “velocity field approach”. They showed that, when the impact velocity is greater than a critical cavitation velocity, the inertia term becomes important in the penetration resistance, where the separation point (between the projectile and the surrounding penetrated metal) moves toward the tip of the projectile nose. However, when the impact velocity is less than the critical cavitation velocity, the inertia term is negligibly small, and only a constant static term exists in the penetration resistance.

It is found that the above studies mainly focused on metal targets. However, the two-phase feature of the deceleration history during rigid projectile penetration also exists in the concrete-like brittle targets. This was shown directly in deceleration–time measurements performed by Forrestal et al. [10]. These experimental data demonstrated that the deceleration history with time exhibits a relatively plateaued response. Moreover, a semiempirical prediction which did not consider the dynamic term was in good agreement with the experimental results.

Zhang and Mu [34] adopted the simulation method to investigate the deceleration history of the penetration projectile for concrete-like materials, the quasi-constant feature of the deceleration history was also observed. Meanwhile it was found that the friction between projectile nose and target has significant effect on the penetration depth in the tunneling stage of the penetration.

It is also noted that the discussion held by Kong et al. and Rosenberg and Dekel [26,27] supported the two-phase feature of the deceleration history. Analytical results from Kong et al. [27] demonstrated that the time-dependent deceleration of projectile during the penetration process was not observed clearly, and the deceleration–time curves of projectile were almost constant up to a certain striking velocity. Yet, when the striking velocity was relatively high, the velocity-dependent deceleration could be clearly obstained.

The aforementioned results substantiate the two-phase behavior of the deceleration history of a penetrating rigid projectile. It is evident from the above dynamic spherical cavity expansion model and velocity field theories, as well as from numerical and experimental studies and from semi-empirical equations, that the projectile deceleration history in the rigid penetration mode can be delineated by two distinct phases. When the initial striking velocity is below a certain critical striking velocity, the projectile axial resistant force shows an approximate peak plateau response; however, when the initial striking velocity exceeds this value, the projectile axial resistance force presents a well-defined peak. Furthermore, the event is not only found in metal-like materials, but also in concrete-like materials. This proves that the two-phase phenomenon is not an accidental event, whereby it does exist in the rigid penetration process, which is the analytical premise for the present paper.

Note that the discussions above mainly focused on the two-phase feature, while the velocity-independent characteristic was not discussed, as mentioned in the research and development (R&D) approach [20].

### 2.2. Role of SSF in Two-Phase Penetration Behavior

As stated above, it is acceptable that projectile deceleration exhibits two-phase behavior in rigid penetration for concrete-like materials, as demarcated by a critical striking velocity. The two corresponding phases were identified as the “squeeze and push” (SP) penetration phase and the Dynamic Spherical Cavity Expansion (DSCE) penetration phase [6], according to the different features.

The SP phase mainly involves the quasi-constant behavior of the deceleration history, which can be expressed by the following equation:

(1) $$\frac{\partial F_{r}}{\partial V}\approx 0$$

where *F*_r_ is the resisting force, acting along the trajectory of the penetrating projectile, and *V* is the instantaneous penetration velocity.

According to the force analysis of the projectile nose in Figure 1, combined with the popular formula of the projectile normal direction stress in the rigid penetration consisting of the quasi-static term and the dynamic term (or the inertia term) [1,2,5,9], the normal stress can be given as

(2) $$\sigma_{n}=Af_{c}+f(V)$$

where *A* is a dimensionless coefficient, and $f_{c}$ is the target yielding stress. Note that the dynamic term f(V) does not give a specific function. Finally, using Equation (1), Fr can be deduced with the following equation:

(3) $$\frac{\partial \mu }{\partial V}\left[Af_{c}\beta_{1}+\beta_{1}f(V)\right]\approx -\frac{\partial f(V)}{\partial V}(1+\beta_{1}\mu )$$

Note that, in the analysis process, the surface sliding friction coefficient μ is assumed to be a function of penetration velocity. Consequently, the following equation is gained by integrating Equation (3):

(4) $$F_{r}=\frac{\pi d^{2}}{4}\left(Af_{c}+Af_{c}\beta_{1}\mu_{s}\right)$$

The detailed analytical process was described in [6], where *d* is projectile diameter, and β_1_ and μs are the model coefficient related to the projectile shape and the static friction coefficient. 

Equation (4) indicates that the axial resisting force includes two parts: the quasi-static term $Af_{c}$ and frictional term $Af_{c}\beta_{1}\mu_{s}$. In addition, and more importantly, there is a vanishing of the dynamic term f(V).

Apparently, Equation (4) indicates that the friction has an effect on this SP phase. The driving mechanism behind the quasi-constant behavior can be attributed to two actions: one is the “push” action since the existence of the penetration velocity; the other is the “squeeze” action. The premise of Equation (4) is that $\partial F_{r}/\partial V$ is approximately equal to zero; however, in the real situation, f(V) still exists as a minimal contribution. Similarly to Warren’s comments [23] stating that the strength term (named the quasi-static term in the present paper) overshadows the inertial term (the dynamic term in Equation (2)), the target inertia term can be neglected. In terms of the dynamic cavity expansion, it can be explained that, in the finite penetration time, the dynamic cavitation expansion velocity is low such that the cavitation diameter is always smaller than the projectile diameter during the penetration process. Consequently, the “squeeze” action is caused upon accompanying the SSF. Eventually, the “push” action and the “squeeze” effect collectively contribute to the occurrence of the deceleration–time quasi-constant phenomenon.

**Figure 2:**
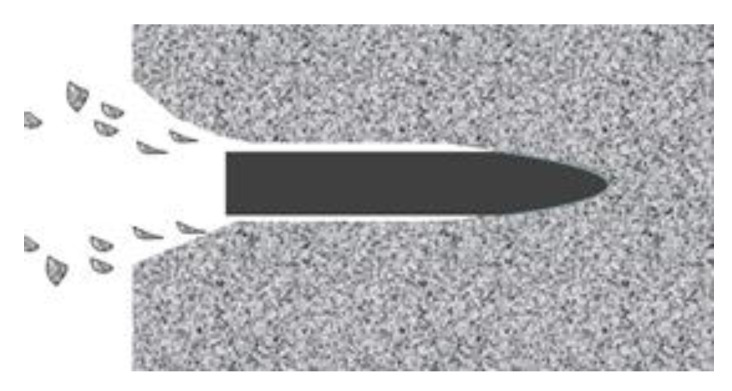
Cavity expansion process.

Furthermore, with the striking velocity increasing, the DSCE mechanism gradually dominates the penetration process since the cavity diameter finally exceeds the projectile diameter in the finite penetration time in Figure 2. The accompanying phenomenon is that the “squeeze” effect vanishes. Therefore, the chained result is that the SSF between projectile and target can be neglected. In this phase, the corresponding dynamic term can be expressed as Equation (5) according to the dynamic spherical cavitation expansion theory by Luk and Forrestal [11,17]. The corresponding projectile resistant force is popular, as shown in Equation (6) [11,17,27,28].

(5) $$f(v)=B\rho v^{2}$$

(6) $$F_{r}=\frac{\pi d^{2}}{4}(Af_{c}+BN^{*}\rho V^{2})$$

Eventually, it can be concluded that the role exchange between the SSF and the DSCE contributes to the two-phase behavior. SSF plays a major role in characterizing the SP phase, which can be denoted as an “external mechanism” since it can be changed by enhancing the projectile lubrication ability. Moreover, in this phase, the DSCE mechanism still exists, but the effect is relatively weak such that it can be neglected, while the quasi-static term and SSF term together contribute to the quasi-constant behavior of the deceleration history, where, according to the definition of the SSF, the quasi-static term can be denoted as an “internal mechanism”. In the DSCE phase, a well-defined peak performance of the deceleration history is found, accompanying the disappearance of the quasi-constant phenomenon; thus, Equation (4) considering the friction effect is not available to predict the penetration depth. The other internal mechanism DSCE is activated totally to govern the penetration process, and the corresponding cavity beyond the projectile diameter is generated since in the finite penetration time the cavity expansion size exceeds the projectile diameter. Consequently, this results in the disappearance of the “squeeze” effect.

**Figure 3:**
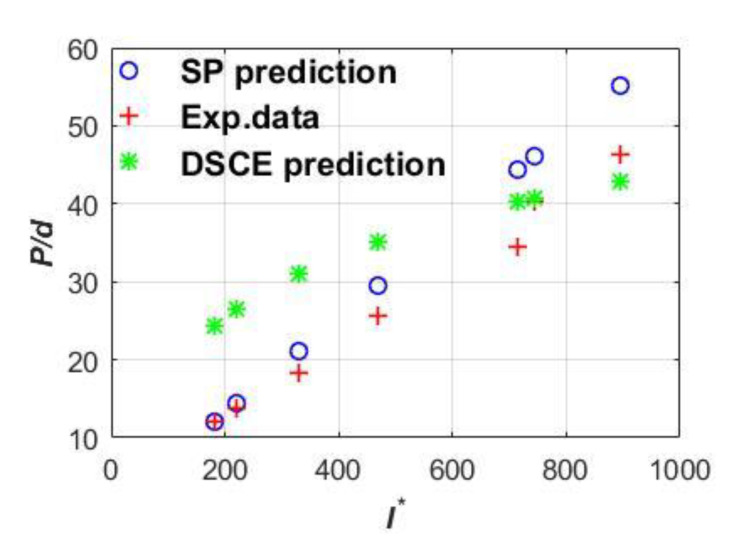
Typical comparison results of long shank projectile targets.

**Figure 4:**
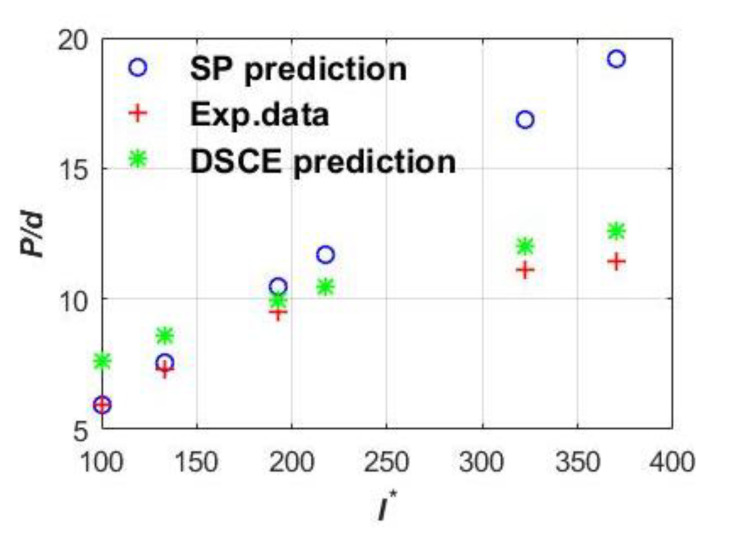
Typical comparison results of short shank projectile targets.

The feasibility of two-phase penetration analysis was validated by comparisons with 12 penetration experimental cases [6]. Two typical results are given in Figure 3 and Figure 4, where $I^{*}$($MV_{s}^{2}/d^{3}f_{c}$) is one dimensionless parameter related to the striking velocity and, similarly, the corresponding vertical coordinate *P/d* is also dimensionless as a function the unit of the penetration depth and the projectile diameter. Note that Figure 4 employs the short shank projectile [35]. Clearly, Figure 3 and Figure 4 show the two-phase characteristic SP prediction has good agreement when considering low striking velocity. With the increase in striking velocity, the DSCE prediction gradually approaches the experimental data, eventually dominating the phase of high striking velocity. 

## 3. Numerical Simulation Investigations into the Role of Friction

The two-phase analyses in Section 2 strongly demonstrate that the SSF plays an important role in the SP phase. To further substantiate the role of SSF in the two-phase penetration behavior, a series of numerical simulations using Autodyn software were performed (Autodyn software is a uniquely versatile explicit analysis tool for modeling the nonlinear dynamics of solids, fluids, gas, and their interaction), where typical Hanchak perforation experiments with 48 MPa concrete [36] and Forrestal penetration experiments with 51 MPa concrete [12] were chosen. 

### 3.1. Simulation Approach for Perforation and Penetration Experiments

**Figure 5:**
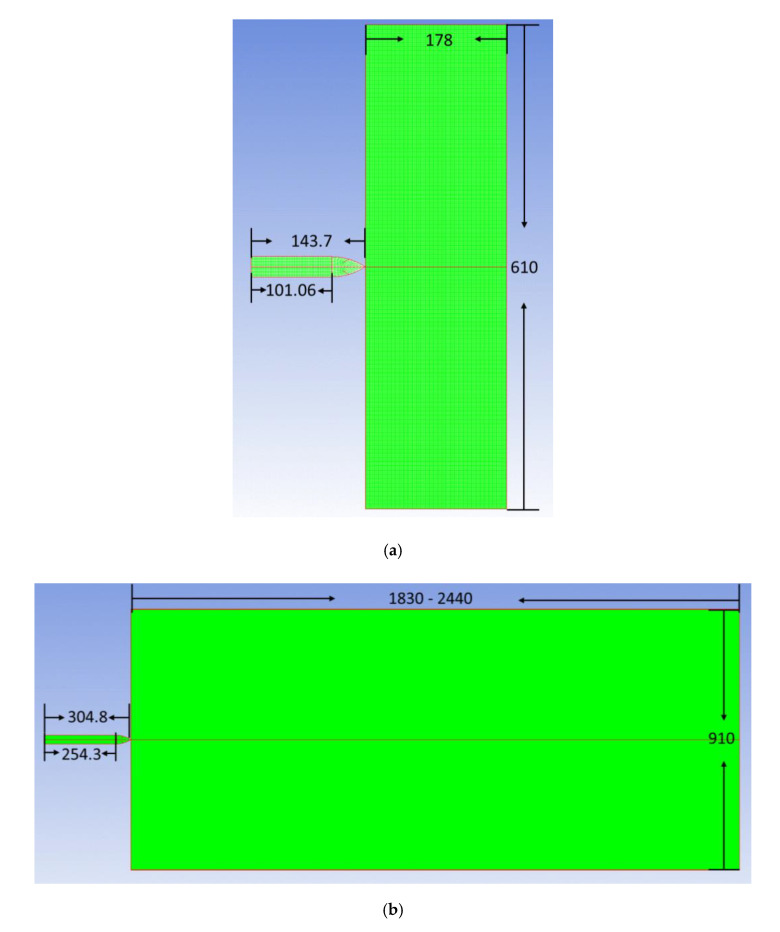
Numerical simulation configuration: (**a**) perforation experimental configuration; (**b**) penetration experimental configuration.

All simulations in the study were performed with the Autodyn-2D code (version 11.0) [37], using the Lagrange processor. Moreover, aiming to assure that the projectile maintains the rigid during the penetration process, its strength was artificially set to a relatively high value of 20 GPa for all the cases. Two geometrical configurations corresponding to the perforation and penetration experiments were used, as shown in Figure 5a,b, where the mesh size was set to 1 mm × 1 mm. For the penetration experiments, the target thickness changed with the different striking velocity. Table 1 lists each target thickness, where a 2 mm steel culvert was added as the boundary condition on the basis of the penetration experimental configuration.

### 3.2. Constitutive Models of Concrete-Like Materials

To our knowledge, in order to count on the simulation results, the rigorous material models that properly represent the material behavior is required. The current paper aims to clearly study the SSF effect to the penetration process, the piece-wise linear Drucker‐Prager concrete material model [37] and the P‐α EOS (Equation of State) [38] were used to simulate the corresponding perforation and penetration experiments. The feasibility of the material models and the corresponding parameters was proven by studying the uniaxial dynamic compressive behavior of concrete-like materials [39], where it was focused on resolving questions associated with the compressive strength enhancement under the increased strain-rate. Since the material models are not the focus in this paper, more details on these models have been given in Reference [39]. The corresponding material parameters are listed in Table 2.

### 3.3. Simulation Results and Discussions 

#### 3.3.1. Perforation Cases without Considering the SSF

The SSF between projectile and target is a complicated problem, and there is still no feasible method for in situ measurements during the penetration process. Normally, a specific and accurate algorithm is requested to reproduce the friction physics for the numerical method; however, this is beyond the scope of this paper. Our main purpose was to qualitatively and numerically investigate the role of SSF in rigid penetration into concrete-like material targets.

**Figure 6:**
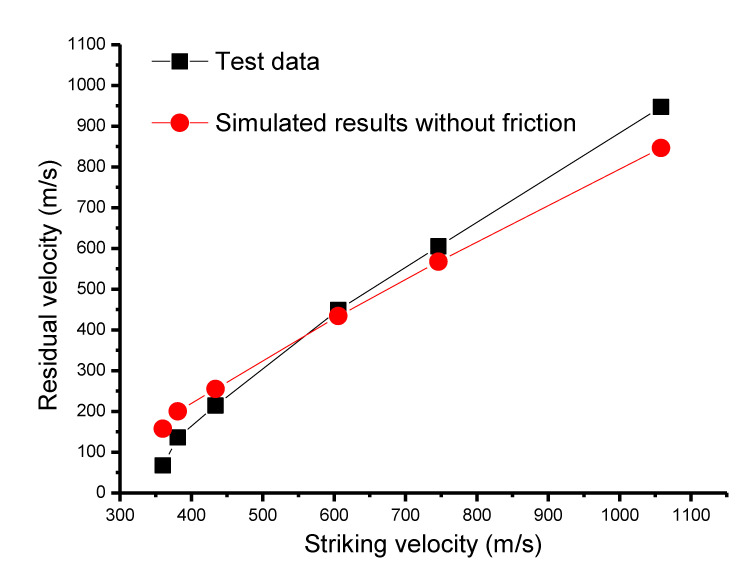
Perforation comparison results between experimental data and simulated results without considering the surface sliding friction (SSF).

Firstly, residual velocity comparisons were conducted between perforation experimental data and simulated results without including the friction, as given in Figure 6. Interestingly, it was found that there exists a demarcation point for the striking velocity. Below the demarcation point, the simulated results were all beyond the experimental data. Conversely, numerical results were less than the corresponding experimental data when the striking velocity was above the demarcation point. 

Aiming to investigate the role of SSF, the simulation results above were analyzed on the basis of the following error comparisons of residual velocity.

At the beginning, it was postulated that the friction always existed in all experimental cases. The simulated cases including the SSF and without regarding the SSF are denoted as Sf≠0 and Sf=0, respectively. The corresponding experimental results are denoted as *E*. According to the assumption of the existence of friction, the following equation was obtained:

Sf ≠ 0-E < Sf = 0-E(7)

Furthermore, since the SSF mainly retards the penetration of a rigid projectile into the target, it could be gained that Sf ≠ 0 < Sf = 0. Therefore, this indicates that the simulated results for the cases below the demarcation point were consistent with the postulation of SSF existence. 

On the other hand, for the shots beyond the demarcation point, it was found that Sf = 0 was smaller than the experimental data. According to the SSF retarding feature with respect to the penetration, the following logic relation was obtained:

Sf ≠ 0 < Sf = 0 < E(8)

which is contradictory with the postulation of SSF existence. This is evidence to support that SSF may be negligible for shots beyond the demarcation point. Consequently, it could be concluded that the SSF effect was phased, mainly depending on a certain critical striking velocity, which is consistent with the two-phase analyses in Section 2. 

#### 3.3.2. Penetration Cases without Considering the SSF

**Figure 7:**
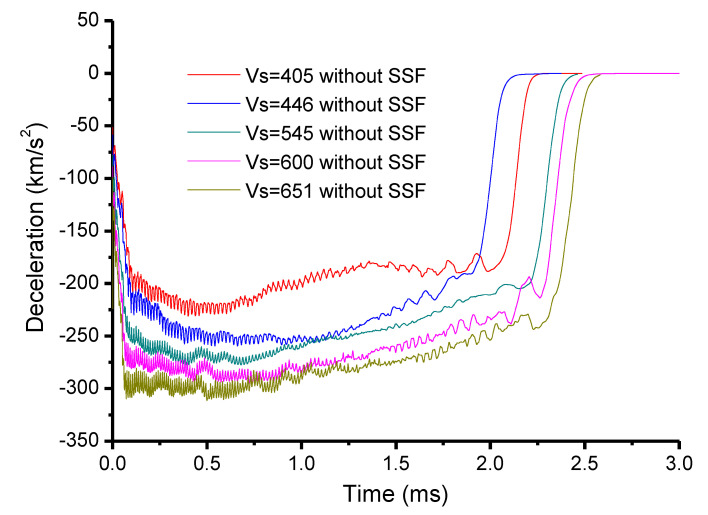
Penetration simulations without SSF at a relatively low striking velocity.

**Figure 8:**
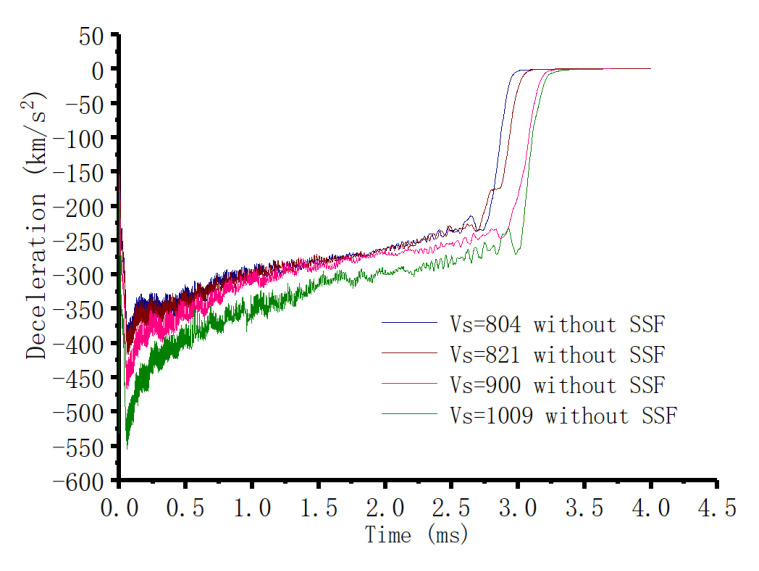
Penetration simulations without SSF at a relatively high striking velocity.

Figure 7 and Figure 8 give the simulation results for the penetration cases without considering the SSF. Similarly, a two-phase phenomenon was obtained for the deceleration history. When the initial striking velocity was below 804 m/s, the deceleration history exhibited a mild plateaued response, and the response increased with the rise in striking velocity. For the cases beyond 804 m/s (including the case with 804 m/s), a well-defined peak of the acceleration history was gained. As before, the increase in striking velocity contributed to a rise in the corresponding peak, which again conformed to the two-phase behavior of the deceleration history in Section 2.1. On the other hand, Figure 7 demonstrates that the response of the deceleration history was sensitive to the striking velocity for the first phase of the mild plateau, which is unlike the SP phase in Section 2.2. This directly led to an association with the SSF since it was not taken into account in the penetration simulations. Thus, it was indicated that SSF can result in an increase in the deceleration, and it behaves as a velocity-dependent feature. Moreover, the comparisons of the penetration depth displayed the same developing trend as the perforation cases. Therefore, it could be concluded that the SSF of the penetration cases also had a critical striking velocity to segment the SSF effect. 

#### 3.3.3. Numerical Simulations Including the SSF

The simulations above adopted an indirect method, which was not strong enough to highlight the SSF effect. Therefore, to further directly elucidate the role of SSF, the corresponding cases considering friction were simulated, as detailed in this section.

**Figure 9:**
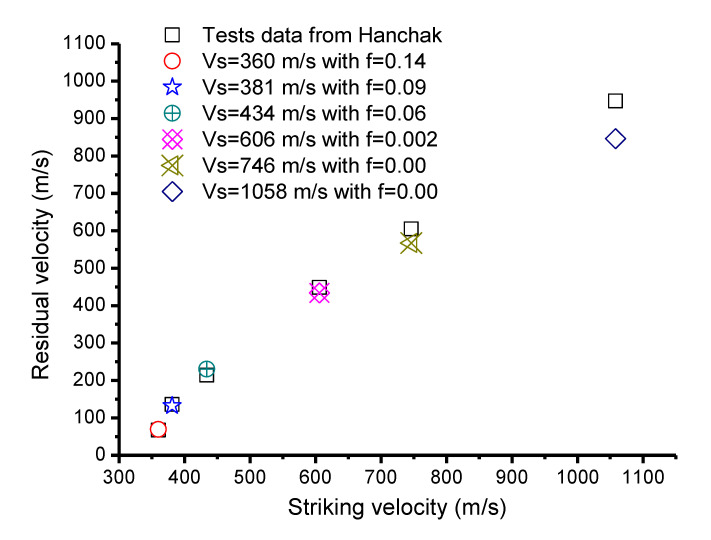
Comparisons of Hanchak perforation experiments considering friction.

As mentioned before, for the SSF coefficient, it is difficult to give a precise algorithm due to the complexity and the measured difficulty during the penetration process; however, a proper static friction coefficient can be identified as the average of SSF [5]. Thus, the static friction coefficient was used to reproduce the experimental data. Figure 9 gives the simulation results of the perforation cases including the friction. It was found that the method of setting the coefficient artificially led to similar results to Figure 6, where shots with 360 m/s, 381 m/s, and 434 m/s could be allocated good values of the friction coefficient close to the experimental data. However, for the other shots, only the friction coefficient of zero fit best with the experiments. This proves that the error in experimental data for the relatively high striking velocity was not due to the SSF, but to other factors.

**Figure 10:**
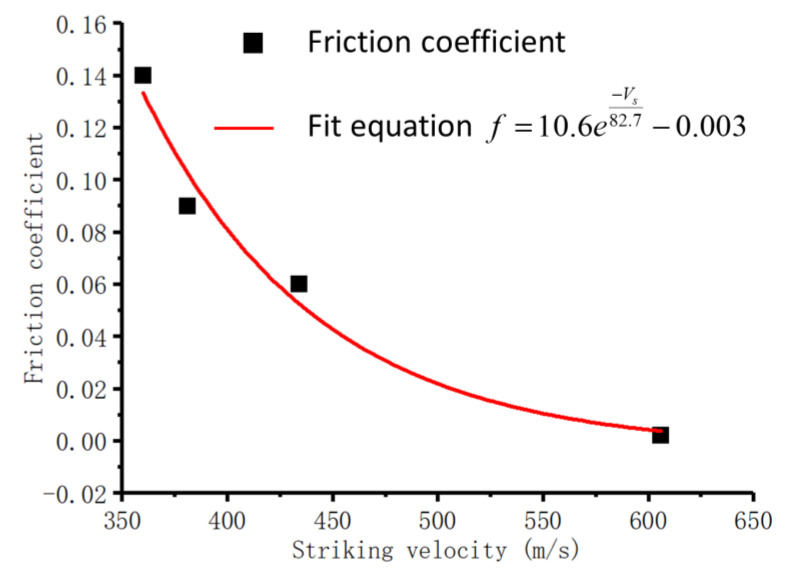
Fit curve of perforation cases.

Meanwhile, as a function of the fit between the static friction coefficient and the initial striking velocity, an exponential function was gained as shown in Equation (9) and Figure 10, which is consistent with the SSF [14], whereby both adopted the exponential function to calculate the corresponding coefficient. To some extent, this implies that using the static friction coefficient as a substitute for the real friction situation is a simple and relatively validated method.

(9) $$f=10.6e^{\frac{-V_{s}}{82.7}}-0.003$$

**Figure 11:**
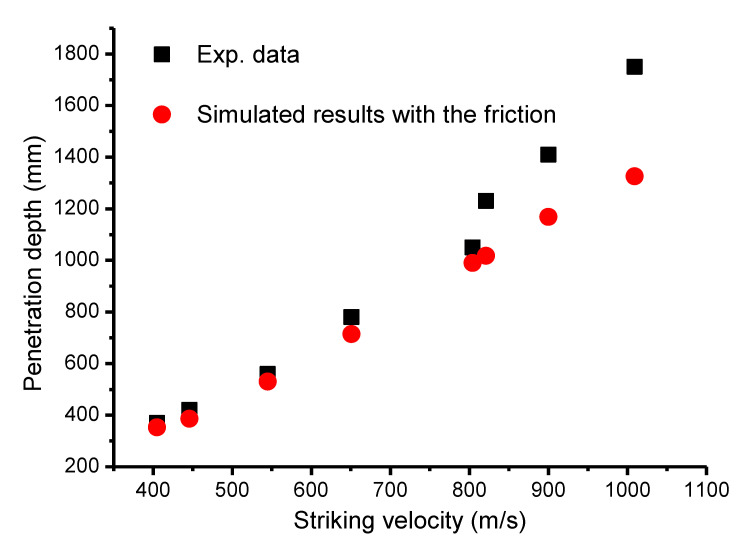
Penetration depth comparisons between experimental data and simulated results.

Subsequently, for the penetration experiments, the static friction coefficient could be derived using Equation (9), as listed in Table 3. With the gained friction coefficient, comparison results with the experimental data are given in Figure 11. Similar characteristics to the perforation experiment were observed. Shots with 405 m/s, 446 m/s, 545 m/s, and 651 m/s showed good agreement with the experimental data. However, above 651 m/s, the simulation results with a friction coefficient of zero derived from Equation (9) were always lower than the experiment data. These results were similar to the cases without considering the friction.

#### 3.3.4. Investigations for Shank Friction Effect 

Note that the analyses above assumed that the main assistant force was from the projectile nose, whereas the effect of projectile shank could be neglected, which is commonly accepted by most researchers [1,2,3,5,15,18].

**Figure 12:**
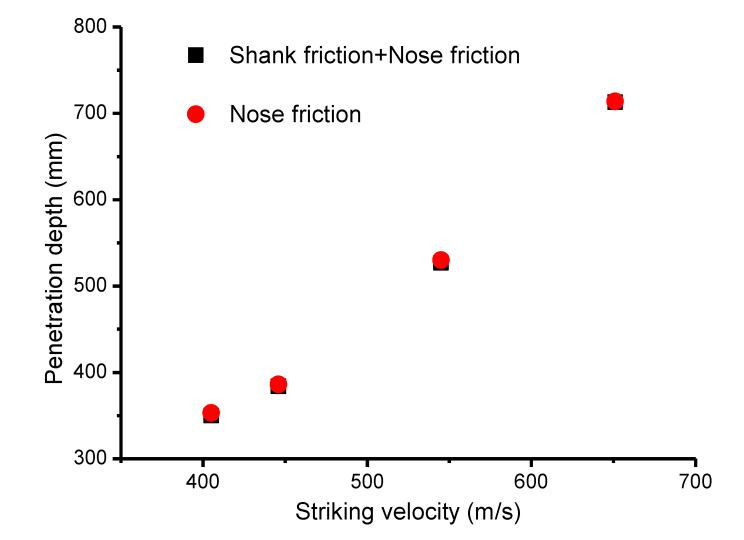
Comparisons of penetration depth between cases considering shank friction and cases not considering shank friction.

**Figure 13:**
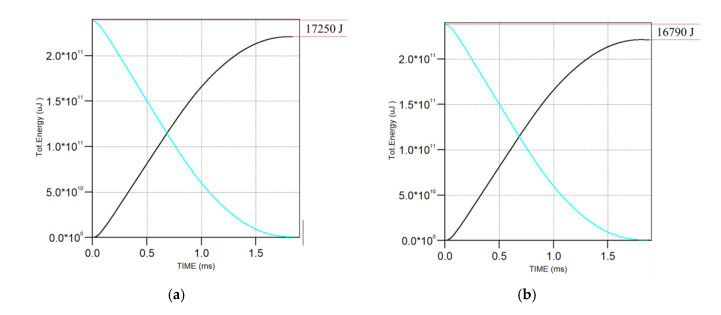
Energy change history of $V_{s}$ = 545 m/s for the projectile and target: (**a**) nose + shank friction; (**b**) nose friction (the blue line and black line denote the projectile energy and the target energy, respectively).

In this section, the shank friction effect was revised numerically. Figure 12 gives the comparison results with the shank friction effect. The comparison results indicated that, in the SP phase, the corresponding penetration depth was almost same as in the cases without considering the shank friction, where the maximum error was 8 mm due to the addition of the shank friction. Moreover, it was found that the corresponding energy loss of the projectile for nose friction cases and nose + shank friction cases had a weak variation below 3%. Figure 13 gives the energy time history with $V_{s}$ = 545 m/s, where it was found that the addition of the shank friction only resulted in a further 2.7% energy loss, such that the effect could be deemed negligible.

**Figure 14:**
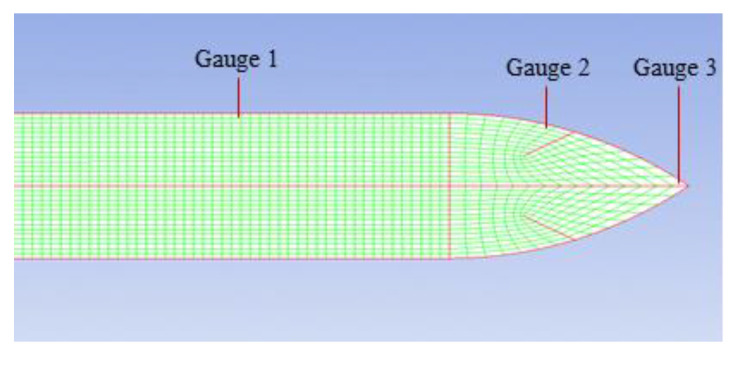
Positions for three gauges.

**Figure 15:**
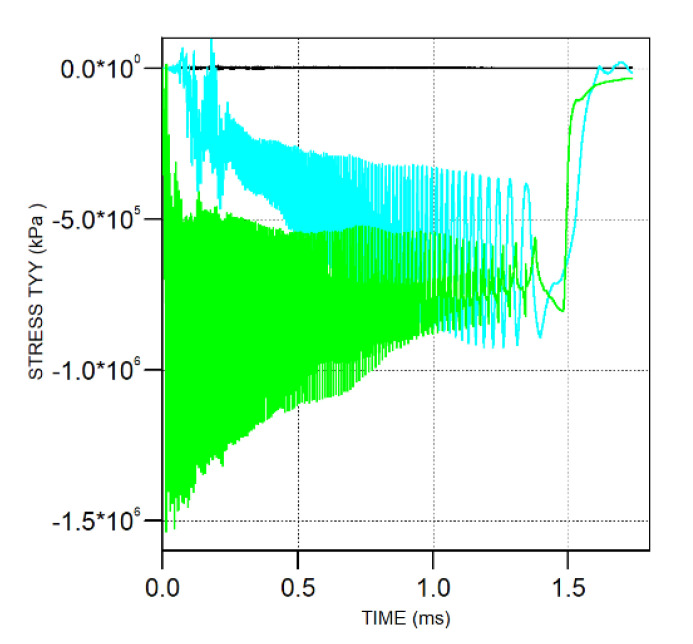
Time history of the stress perpendicular to the axial orientation for the three gauges (black line = Gauge 1, blue line = Gauge 2, and green line = Gauge 3).

On the other hand, aiming to further prove the shank friction effect, the surface stress of the projectile was investigated. Three gauges were selected as given in Figure 14. The stress history (in Figure 15) perpendicular to the axial orientation shows that Gauge 1 in the shank part did not clearly exhibit a stress response, with a corresponding value of almost zero. Conversely, Gauge 2 and Gauge 3 in the nose part demonstrated a relatively large stress response. Thus, clearly, the stress changes for three typical gauge points indicated that, in the penetration process, the surface contact force between the shank and the target was very slight such that it could be deemed negligible.

### 3.4. Discussions of SSF Role 

Eventually, according to the numerical simulations of the perforation and penetration cases, it could be summarized that the role of SSF is phased. At the range below a certain critical striking velocity, adding the friction could reproduce the experimental data; when exceeding the critical striking velocity, the simulated results without considering the friction were closest to the experimental data. Furthermore, the critical striking velocity for the penetration experiment was above 651 m/s, which is consistent with the 703 m/s gained according to the two-phase prediction models in Section 2. Simultaneously, associated with the deceleration history in Figure 7 and Figure 8, it could be judged that the two-phase characteristic in the simulations was consistent with the two-phase penetration feature. 

The comparisons of penetration depth, energy loss, and surface contact force between the projectile shank and the target indicate that the effect of the shank friction could be deemed negligible, which further proves the reasonability of the present analytical work. 

Combined with the two-phase analytical results in Section 2, the SSF phased feature was confirmed. Below a certain critical striking velocity, it was reasonable to illustrate the quasi-constant phenomenon of the deceleration history by considering the SSF, where the DSCE mechanism could be ignored. In the range of the striking velocity, the interaction between the push effect from the projectile itself and the squeeze effect caused by the contact between the projectile and the target governed the phase. However, with the increase in striking velocity, the increasing amplitude of the deceleration resulting from the SSF became more and more limited, with the simulation results in Figure 14 clearly supporting this point. When the striking velocity was beyond the critical striking velocity, the mechanism governing the penetration process happened to the change, where the DSCE mechanism gradually dominated the penetration. This could be explained by the phase the diameter of the dynamic expansion cavity being larger than the projectile diameter. Consequently, the squeeze effect between the projectile and the target became very slight, such that the effect could be deemed negligible. The result was that the SSF could not be taken into account in the penetration analytical process. The analytical method in Section 2 and the numerical simulations in this section both prove that the sensitivity of SSF to the penetration process is one of the factors driving the two-phase feature. In the phase of the quasi-constant deceleration history, SSF was sensitive to the penetration, where the DSCE mechanism (dynamic term) shows a weak effect. Oppositely, when entering into the phase of the well-defined peak deceleration history, there was an exchange between the SSF and DSCE mechanisms. The SSF effect became a non-sensitive to the penetration process, whereby the DSCE mechanism was totally activated. These analytical results allow us to better understand the two-phase behavior of the deceleration history, and they will contribute to a simplification of the corresponding equations for penetration depth and residual velocity, which is beneficial to real engineering applications.

There are two points to be taken into consideration for these simulations. One is that, in the perforation simulations, the reinforcing bar was neglected. Sliter [40] indicated that, when the ratio of the reinforcing bar was in the range of 0.3%–1.5%, its effect could be left out. Moreover, Holmquist [41] and Tham [42] proved that the effect of the reinforcing bar in the Hanchak perforation experiment is very slight. The other point is that, for the penetration simulation, the 600 m/s shot was added to highlight the change in deceleration history; however, this was not performed in the experiment [12]. 

## 4. Remarks and Conclusions

The study of penetration process analyses and numerical simulations illustrated the role of SSF in the two-phase feature of the deceleration history. These results provide insight into the major role of SSF in characterizing the SP phase of two-phase penetration. SSF and the quasi-static term both contribute to the quasi-constant behavior of the deceleration history for nondeforming projectile penetration into concrete targets. The two-phase penetration analyses and numerical simulations both showed that considering the SSF could allow an accurate prediction of the penetration depth below a certain critical striking velocity. With the rise in initial striking velocity, the penetration would enter into the DSCE phase, where the SSF effect is weak such that it can be ignored, while the DSCE is totally activated. Meanwhile, the numerical simulation results indicated that a friction coefficient of zero fit best with the experiments in the DSCE phase, further supporting the role exchange between SSF and DSCE during the penetration process. Last but not least, the variation of penetration depth, energy loss of the projectile, and the stress features of the surface gauge in the shank part and the nose part all indicated that the shank friction has a very slight effect such that it can be deemed negligible, where it was found that the addition of the shank friction only resulted in an extra 2.7% energy loss for the 545 m/s shot. This further validates the reasonability of the present study. 

It is necessary to emphasize that, in two-phase penetration analyses, the axial resistant force of the projectile nose is not needed to deduce the relative complex sliding friction, and only the static friction coefficient is required. More importantly, this point is obtained from the analysis itself, independent of any assumption. Furthermore, the analytical process considers the SSF feature depending on the striking velocity. The increase in “push” effect resulting from the increase in striking velocity contributes to a decrease in the “squeeze” effect due to the decrease in SSF sensitivity. This was consistent with the SSF feature depending on the striking velocity. The simulation results in Section 3 also support the interaction between “push” and “squeeze”, where the deceleration history showed a rising trend when not considering the friction, indicating that the SSF is the main driving mechanism for the quasi-constant behavior of the deceleration history, independent of the striking velocity.

This paper analytically and numerically investigated the role of SSF in the two-phase behavior of the deceleration history. This phased SSF feature improves the understanding of the corresponding two-phase penetration response. The study in this paper is beneficial for accurately predicting the penetration depth and the residual velocity in real engineering applications. 

## Figures and Tables

**Table 1:** Thickness corresponding to different striking velocity in tests.

$V_{s}\;m/s$	405	446	545	651	804	821	900	1009
H mm	1830	1830	2130	2130	2130	2130	2440	2440

**Table 2:** Material parameters involved in the simulations

Parameters for EOS						Basic Properties for Materials	
Solid density $\rho_{si}$	2.684 × 10^3^ kg/m^3^	Solid compaction pressure $P_{lock}$	6000 MPa	*B_0_*	1.22	Uniaxial-compressive strength	48 MPa
Initial Porous density $\rho_{0}$	2.314 × 10^3^ kg/m^3^	*A_1_*	3.53 × 10^4^ MPa	*B_1_*	1.22	Hydro tensile limit	4 MPa
Initial sound speed $C_{0}$	2.92 × 10^3^ m/s	*A_2_*	3.96 × 10^4^ MPa	*n*	3	Shear Modulus	14.88 GPa
Initial compaction pressure $P_{crush}$	23.3 MPa	*A_3_*	9.04 × 10^3^ MPa			Poisson’s ratio *r*	0.2

**Table 3:** Friction coefficients corresponding to the striking velocity of penetration tests.

$V_{s}\;m/s$	405	446	545	600	651
f	0.08	0.045	0.01	0.002	0.001
